# *Selaginella* *tamariscina* Attenuates Metastasis via Akt Pathways in Oral Cancer Cells

**DOI:** 10.1371/journal.pone.0068035

**Published:** 2013-06-14

**Authors:** Jia-Sin Yang, Chiao-Wen Lin, Chung-Han Hsin, Ming-Ju Hsieh, Yu-Chao Chang

**Affiliations:** 1 Institute of Medicine, Chung Shan Medical University, Taichung, Taiwan; 2 Institute of Oral Sciences, Chung Shan Medical University, Taichung, Taiwan; 3 Department of Dentistry, Chung Shan Medical University Hospital, Taichung, Taiwan; 4 School of Medicine, Chung Shan Medical University, Taichung, Taiwan; 5 Department of Otolaryngology, Chung Shan Medical University Hospital, Taichung, Taiwan; 6 School of Dentistry, Chung Shan Medical University, Taichung, Taiwan; China Medical University, Taiwan

## Abstract

**Background:**

Crude extracts of *Selaginella* *tamariscina* , an oriental medicinal herb, have been evidenced to treat several human diseases. This study investigated the mechanisms by which *Selaginella* *tamariscina* inhibits the invasiveness of human oral squamous-cell carcinoma (OSCC) HSC-3 cells.

**Methodology/Principal Findings:**

Herein, we demonstrate that *Selaginella* *tamariscina* attenuated HSC-3 cell migration and invasion in a dose-dependent manner. The anti-metastatic activities of *Selaginella* *tamariscina* occurred at least partially because of the down-regulation of matrix metalloproteinases (MMP)-2 and MMP-9 gelatinase activity and the down-regulation of protein expression. The expression and function of both MMP-2 and MMP-9 were regulated by *Selaginella* *tamariscina* at a transcriptional level, as shown by quantitative real-time PCR and reporter assays. Chromatin immunoprecipitation (ChIP) data further indicated that binding of the cAMP response element-binding (CREB) protein and activating protein-1 (AP-1) to the MMP-2 promoter diminished at the highest dosage level of *Selaginella* *tamariscina* . The DNA-binding activity of specificity protein 1 (SP-1) to the MMP-9 promoter was also suppressed at the same concentration. *Selaginella* *tamariscina* did not affect the mitogen-activated protein kinase signaling pathway, but did inhibit the effects of gelatinase by reducing the activation of serine–threonine kinase Akt.

**Conclusions:**

These results demonstrate that *Selaginella* *tamariscina* may be a potent adjuvant therapeutic agent in the prevention of oral cancer.

## Introduction

Head and neck squamous-cell carcinoma accounts for approximately 3% of all cancers in the United States, and oral squamous-cell carcinoma (OSCC) is the most common form of head and neck cancer [1]. The high rate of metastasis to cervical lymph nodes causes the poor survival rate of oral cancer [2]. Cancer cells typically spread by secreting various molecules that degrade the extracellular matrix (ECM), invading the blood vessels, and migrating to distant organs [3]. Matrix metalloproteinases (MMPs) are a major group of enzymes that regulate ECM composition during normal development and pathological responses [4]. Although various MMPs contribute to cancer cell metastasis, the gelatinases MMP-2 and MMP-9 have been most intensively studied [5]. MMP-2, also known as gelatinase A, is a 72-kDa protein expressed in most tissues and cells [6]. In contrast, MMP-9 (Gelatinase B), a 92-kDa protein, is conditionally observed in leukocytes [7]. Elevated MMP-2 and MMP-9 expression have been observed in invasive and metastatic cases of human oral cancer [8–10]. Hence, concentrated efforts have been made to develop MMP inhibitors (MMPIs) to halt the spread of cancer cells [11].

*Selaginella* *tamariscina* is an herb traditionally used in oriental medicine that exhibits several therapeutic abilities. First, because *Selaginiallatamariscina* has been shown to reduce blood sugar and serum lipid peroxide levels, it exhibits potential uses in the treatment of diabetes [12,13]. Second, bioflavonoids isolated from *Selaginella* *tamariscina* demonstrated antibacterial and antifungal effects [14–16]. Third, crude extracts from *Selaginella* *tamariscina* have inhibited human mesangial cell proliferation, and have decreased interleukin-1beta and tumor necrosis factor-alpha production [17]. Fourth, *Selaginella* *tamariscina* could be a potential chemopreventive agent against various human cancer cell lines, such as gastric cancer [18], lung cancer [19], breast cancer [20], and cervical cancer [21]. The aim of this study was to elucidate the effects of *Selaginella* *tamariscina* on human OSCC HSC-3 cells. Our results showed that *Selaginella* *tamariscina* halted oral cancer cell migration through the down-regulation of MMP-2 and MMP-9 expression and by decreasing DNA-binding activity to promoter elements. In addition, the anti-metastatic effects were associated with the inactivation of serine–threonine kinase Akt.

## Materials and Methods

### Extract from *Selaginella* *tamariscina*

*Selaginella* *tamariscina* was purchased from herb stores and dried whole plants (100 g) were extracted twice with 500 ml of 50% ethanol in distilled water. The pooled extracts were filtered and concentrated at 70°C using a rotary evaporator under low pressure. The concentrated crude extract was frozen at −80°C for 2-3 days and then it was freeze-dried in a lyophilizer and stored at −20°C. The extraction yield was 2.8% (w/w) and the chemical profile of Selaginella tamariscina extract (STE) was analyzed by using high-pressure liquid chromatograms (HPLC)-mass spectrometer [19]. Briefly, *Selaginella* *tamariscina* were analysed by HPLC-mass spectrometer using a HPLC (Hitachi L-6200 with an L-4500 Diode Array detector) with a PE Sciex Qstar Pulsar ESI-TOF mass spectrometer. Samples (10 µl) were injected onto a Merck LiChrospher 100 RP-18 column (4 x 250 mm). The column was equilibrated in 0.05% acetic acid/water (solution A) and elution of the components was achieved by increasing the concentration of solution B (100% acetonitrile) from 0 to 100% in 30 min at a flow rate of 1 ml/min. Absorbance was monitored at 254 nm. The molecular masses of the peaks were determined from electrospray ionisation mass spectra using multiply-charged ion profile [19]. The extract was dissolved in dimethyl sulfoxide (DMSO) (Sigma Co., USA) and was prepared at different concentrations for the subsequent experiments.

### Cell culture and *Selaginella* *tamariscina* extract (STE) treatment

HSC-3, a human tongue squamous cell carcinoma cell line obtained from ATCC (Manassas, VA, USA), was cultured in Dulbecco’s modified Eagle’s medium (Life Technologies, Grand Island, NY, USA), 10% fetal bovine serum (Hyclone Laboratories, Logan, UT, USA), 2 mM glutamine, 100 U/mL penicillin, and 100 µg/mL streptomycin. All cell cultures were maintained at 37 ^o^C in a humidified atmosphere of 5% CO_2_. For STE treatment, appropriate amounts of stock solution of STE were added into culture medium to achieve the indicated concentrations and then incubated with cells for indicated time periods, whereas dimethyl sulfoxide solution without STE was used as blank reagent.

### Determination of cell viability (MTT assay)

For cell viability experiment, a microculture tetrazolium (3-(4,5-dimethylthiazol-2-yl)-2,5-diphenyltetrazolium bromide) colorimetric assay was performed to determine the cytotoxicity of STE. HSC-3 cells were seeded in 24-well plates at a density of 5 x 10^4^ cells/well and treated with STE at a concentration between 0–100 µg/mL at 37 ^o^C for 24 h. After the exposure period, the media was removed, and cells were washed with phosphate buffered saline (PBS) and then incubated with 20 µL MTT (5 mg/mL) (Sigma chemical Co., St. Louis, MO, USA) for 4 h. The viable cell number per dish is directly proportional to the production of formazan, which can be measured spectrophotometrically at 563 nm following solubilization with isopropanol.

### In vitro wound closure

HSC-3 cells (1×10^5^ cells/well) were plated in 6-well plates for 24 h, wounded by scratching with a pipette tip, then incubated with DMEM medium containing 0.5% FBS and treated with or without STE (0, 25, 50, 75 and 100 µg/mL) for 0, 12 and 24 h. Cells were photographed using a phase-contrast microscope (×100).

### Cell migration and invasion assays

Cell migration and invasion were assayed according to the methods described by Yang et al. [19]. After a treatment with STE (0, 25, 50, 70 and 100 µg/mL) for 24 h, surviving cells were harvested and seeded to Boyden chamber (Neuro Probe, Cabin John, MD, USA) at 10^4^ cells/well in serum free medium and then incubated for 24 hours at 37 ^o^C. For invasion assay, 10 µL Matrigel (25 mg/50 mL; BD Biosciences, MA, USA) was applied to 8 µm pore size polycarbonate membrane filters and the bottom chamber contained standard medium. Filters were then air-dried for 5 h in a laminar flow hood. The invaded cells were fixed with 100% methanol and stained with 5% Giemsa. Cell numbers were counted under a light microscope. The migration assay was carried out as described in the invasion assay with no coating of Matrigel.

### Determination of MMP-2 and MMP-9 by gelatin zymography

The activities of MMP-2 in conditional medium were measured by gelatin zymography protease assays. Briefly, collected media of an appropriate volume (adjusted by vital cell number) were prepared with SDS sample buffer without boiling or reduction and subjected to 0.1% gelatin-8% SDS-PAGE electrophoresis. After electrophoresis, gels were washed with 2.5% Triton X-100 and then incubated in reaction buffer (40 mM Tris–HCl, pH 8.0; 10 mM CaCl_2_ and 0.01% NaN3) for 12 h at 37 ^o^C. Then gel was stained with Coomassie brilliant blue R-250.

### Preparation of total cell lysates

For total cell lysates preparation, cells were rinsed with PBS twice and scraped with 0.2 mL of cold RIPA buffer containing protease inhibitors cocktail, and then vortexed at 4 ^o^C for 10 min. Cell lysates were subjected to a centrifugation of 10,000 rpm for 10 min at 4 ^o^C, and the insoluble pellet was discarded. The protein concentration of total cell lysates was determined by Bradford assay.

### Western blot analysis

The 20 µg samples of total cell lysates or nuclear fractions were separated by SDS-PAGE on 10% polyacrylamide gels and transferred onto a nitrocellulose membrane using the Mini-Protean Tetra Electrophoresis System as described previously [22]. The blot was subsequently incubated with 5% non-fat milk in Tris-buffered saline (20 mM Tris, 137 mM NaCl, pH 7.6) for 1 h to block non-specific binding and then overnight with polyclonal antibodies against MMP-2, MMP-9, TIMP-1, TIMP-2, three MAPKs (ERK 1/2, JNK 1/2 and p38), or Akt with the specific antibodies for unphosphorylated or phosphorylated forms of the corresponding ERK 1/2, JNK 1/2, p38 and Akt. Blots were then incubated with a horseradish peroxidase goat anti-rabbit or anti-mouse IgG for 1 h. Afterwards, signal was detected by using enhanced chemiluminescence (ECL) commercial kit (Amersham Biosciences) and relative photographic density was quantitated by scanning the photographic negatives on a gel documentation and analysis system (AlphaImager 2000, Alpha Innotech Corporation, San Leandro, CA, USA).

### RNA preparation and TaqMan quantitative real-time PCR

Total RNA was isolated from oral cancer cells using Trizol (Life Technologies, Grand Island, NY) according to the manufacturer’s instructions. Quantitative real-time PCR analysis was carried out using Taqman one-step PCR Master Mix (Applied Biosystems). 100 ng of total cDNA was added per 25 µl reaction with MMP-2, MMP-9 or GAPDH primers and Taqman probes. The MMP-2, MMP-9 and GAPDH primers and probes were designed using commercial software (ABI PRISM Sequence Detection System; Applied Biosystems). Quantitative real-time PCR assays were carried out in triplicate on a StepOnePlus sequence detection system. The threshold was set above the non-template control background and within the linear phase of target gene amplification to calculate the cycle number at which the transcript was detected.

### Transfection and MMP-2, MMP-9 promoter-driven luciferase assays

HSC-3 cells were seeded at a concentration of 5 x10^4^ cells per well in 6-well cell culture plates. After 24 h of incubation, pGL3-basic (vector), MMP-2 or MMP-9 promoter plasmid were co-transfected with a β-galactosidase expression vector (pCH110) into cells using Turbofect (Fermentas, Carlsbad, CA). After 12 h of transfection, cells were treated with vehicle or STE (0 or 100 µg/mL) for 24 h. The cell lysates were harvested, and luciferase activity was determined using a luciferase assay kit. The value of the luciferase activity was normalized to transfection efficiency and monitored by β-galactosidase expression.

### Chromatin immunoprecipitation analysis (ChIP)

Chromatin immunoprecipitation analysis was performed as described previously [23,24]. DNA immunoprecipitated with anti-CREB, anti-SP1 or anti c-fos was purified and extracted using phenol-chloroform. Immunoprecipitated DNA was analyzed by PCR or quantitative PCR by using specific primers as described previously [23].

### Statistical analysis

For all of the measurements, analysis of variance followed by Scheffe posteriori comparison was used to assess the differences between control and cells treated with various concentration of STE. A difference at p < 0.05 was considered to be statistically significant and the experiments were repeated three times.

## Results

### Effects of *Selaginella* *tamariscina* on HSC-3 cell viability and motility

**Figure 1:**
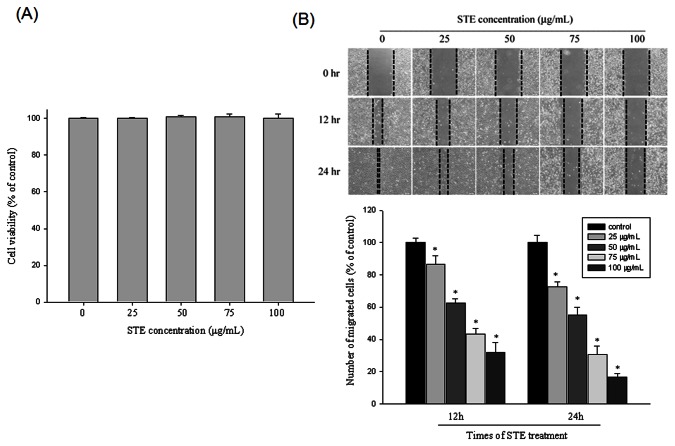
Effect of *Selaginella* *tamariscina* on cell viability and *in* *vitro* wound closure in HSC-3 cells. (A) HSC-3 cells were treated with STE (0, 25, 50, 75 and 100 µg/mL) for 24 h before being subjected to a MTT assay for cell viability. The values represented the means ± SD of at least three independent experiments. (B) HSC-3 cells were wounded and then treated with vehicle (DMSO) or STE (0, 25, 50, 75 and 100 µg/mL) for 0h, 12h and 24 h in 10% FBS-containing medium. At 0, 12 and 24 h, phase-contrast pictures of the wounds at three different locations were taken.

HSC-3 cell viability in the presence of various concentrations (0-100 µg/mL) of *Selaginella* *tamariscina* for 24 hours is shown in Figure 1A. Even the highest concentration, 100 µg/mL, did not have a cytotoxic effect on the HSC-3 cells. We used 0-100 µg/mL of *Selaginella* *tamariscina* to conduct the following experiments. Figure 1B shows the results of using a scratch-wound assay to calculate the migration ability of HSC-3 cells treated with various concentrations of *Selaginella* *tamariscina* . The results demonstrate that *Selaginella* *tamariscina* significantly reduced cell motility both time- and dose-dependently (p<0.001) (Figure 1B and 1C).

### Effects of *Selaginella* *tamariscina* on migration and invasion of HSC-3 cells

**Figure 2:**
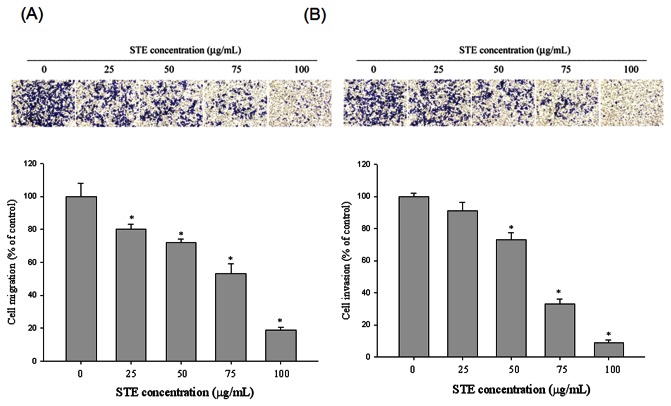
Effect of STE on cell migration and invasion in HSC-3 cells. (A) The cell migration and (B) cell invasion were measured using a Boyden chamber for 16h and 24 h with polycarbonate filters respectively. The migration and invasion abilities of HSC-3 cells were quantified by counting the number of cells that invaded to the underside of the porous polycarbonate as described in the *Materials and Methods* section. The values represented the means ± SD of at least three independent experiments. *p < 0.05 as compared with the vehicle group.

To examine the effects of *Selaginella* *tamariscina* on cell migration and invasion, we used a Boyden chamber assay to detect cell motility. Figure 2A shows that *Selaginella* *tamariscina* significantly inhibited migration in a concentration-dependent manner for 24 hours. Similarly, Figure 2B indicates that the invasiveness of HSC-3 cells was also reduced after incubation with different concentrations (0-100 µg/mL) of *Selaginella* *tamariscina* for 24 hours.

### Effects of *Selaginella* *tamariscina* on MMP-2 and MMP-9 protein expression and enzyme activity

**Figure 3:**
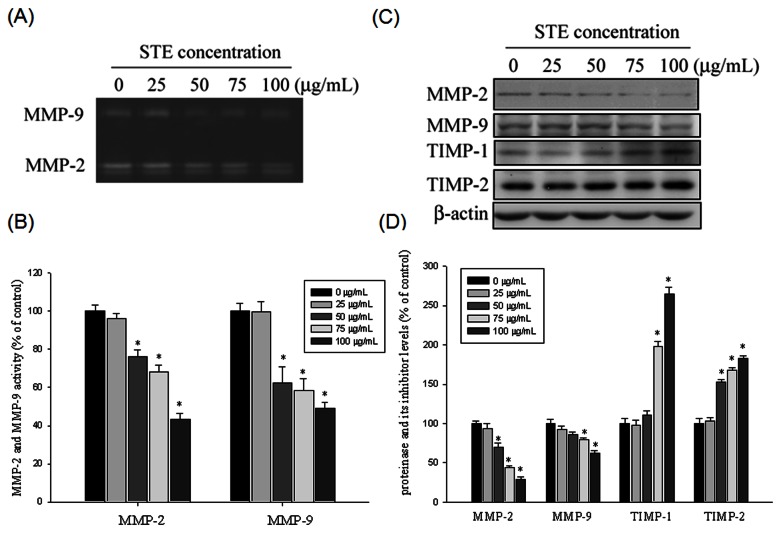
Effects of STE on the activity and protein level of MMP-2, MMP-9 and the protein level of the endogenous inhibitor TIMP-2 and TIMP-1. (A and B) HSC-3 cells were treated with STE (0-100 µg/mL) for 24 h and then subjected to gelatin zymography to analyze the activity of MMP-2 and MMP-9, respectively. (C) HSC-3 cells were treated with STE (0-100 µg/mL) for 24 h and then subjected to western blotting to analyze the protein levels of MMP-2, MMP-9, TIMP-1 and TIMP-2. (D) Quantitative results of MMP-2, MMP-9, TIMP-1 and TIMP-2 protein levels which were adjusted with β-actin protein level. The values represented the means ± SD of at least three independent experiments. *p < 0.05 as compared with the vehicle group.

The ability of *Selaginella* *tamariscina* to suppress the migratory and invasive abilities of HSC-3 cells by decreasing MMP-2 and MMP-9 expression was evaluated using gelatin zymography. Figure 3A shows that the enzyme activity of MMP-2 and MMP-9 was suppressed by *Selaginella* *tamariscina* in a concentration-dependent manner. The highest concentration of *Selaginella* *tamariscina* , 100 µg/mL, inhibited MMP-2 and MMP-9 activity by 57% and 51%, respectively (Figure 3B). *Selaginella* *tamariscina* also substantially reduced MMP-2 and MMP-9 protein expression when detected using western blotting (Figure 3C). Thus, we suggest that the anti-metastatic ability of *Selaginella* *tamariscina* at least partially inhibited MMP-2 and MMP-9 expression. Investigation of the effects of STE on the protein expression of the MMPs endogenous inhibitor, TIMP-1 and TIMP-2, showed that STE induced TIMP-1 and TIMP-2 upregulation in a concentration-dependent manner (Figure 3C and 3D)

### Effects of *Selaginella* *tamariscina* on MMP-2 and MMP-9 mRNA expression and DNA-binding activity

**Figure 4:**
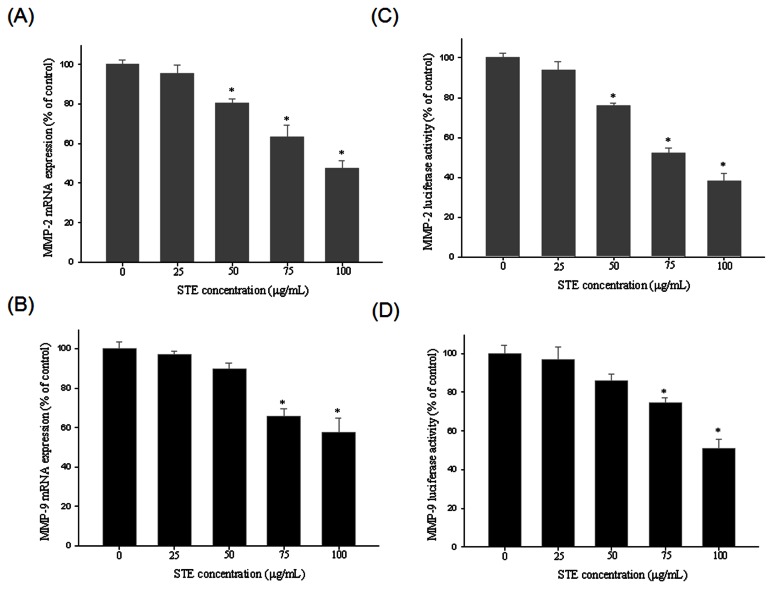
STE suppresses MMP-2 and MMP-9 expression at a transcriptional level.

The effects of *Selaginella* *tamariscina* on MMP-2 and MMP-9 mRNA expression were also examined. A low level of MMP-2 and MMP-9 mRNA expression was observed at the highest dose of *Selaginella* *tamariscina* (100 µg/mL) for 6 hours (Figure 4A and 4B). To further investigate how *Selaginella* *tamariscina* regulates the transcriptional activity of MMP-2 and MMP-9, we conducted a luciferase reporter assay in which both *Selaginella* *tamariscina* and the control cells were transfected with an MMP-2 and MMP-9 promoter construct. Figure 4C shows that the MMP-2 promoter activity was reduced by *Selaginella* *tamariscina* in a dose-dependent manner. Similarly, approximately 50% inhibition of MMP-9 promoter activity was evident at 100 µg/mL of *Selaginella* *tamariscina* (Figure 4D). These observations suggest that *Selaginella* *tamariscina* regulates MMP-2 and MMP-9 activity at the transcriptional level.

HSC-3 cells were treated with STE (0, 25, 50, 75 and 100 µg/mL) for 24 h and then subjected to quantitative real-time PCR to analyze the mRNA expression of MMP-2 (A), or MMP-9 (B). (C) MMP-2 or (D) MMP-9 promoter reporter assay to analyze the promoter activity of MMPs. Luciferase activity, determined in triplicates, was normalized to β-galactosidase activity. The values represented the means ± SD of at least three independent experiments. *p < 0.05 as compared with the vehicle group.

**Figure 5:**
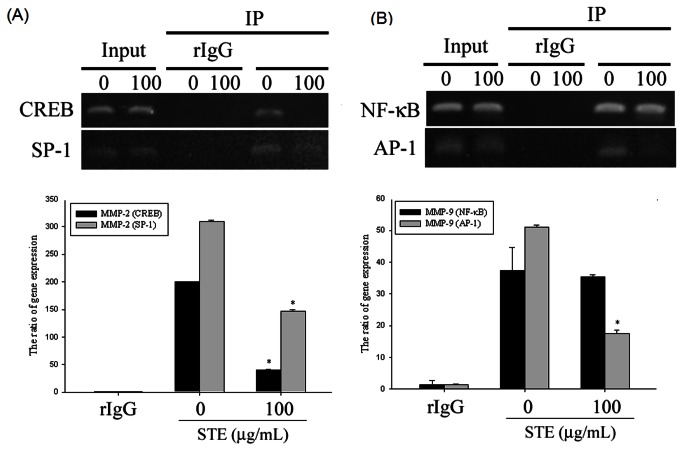
Critical role of transcription factor in STE-induced transcriptional inhibition of MMP-2 and MMP-9 in HSC-3 cells.

Previous studies have shown that MMP promoters are regulated by several transcription factors, such as AP-1, NFκB, CREB, and SP-1 [23,25,26]. We performed a chromatin immunoprecipitation (ChIP) assay to evaluate the involvement of transcription factors in the inhibitory effects of *Selaginella* *tamariscina* on MMP-2 and MMP-9 activity (Figure 5A and 5B). ChIP assay and quantitative real-time PCR showed that *Selaginella* *tamariscina* substantially suppressed binding of CREB and SP-1 to the MMP-2 promoter (Figure 5A). Figure 5B indicates that *Selaginella* *tamariscina* considerably inhibited AP-1, but not the NF-κB DNA-binding to the MMP-9 promoter. These results indicate that *Selaginella* *tamariscina* inhibited MMP-2 and MMP-9 expression by regulating the binding activity of transcription factors on the cis-element of MMP promoters.

HSC-3 cells were treated with STE 100 µg/mL for 24 h and then the nuclear fraction was prepared as described in "*Materials and Methods*". ChIP analysis of the association of various transcription factors with the MMP-2 (A) or MMP-9 (B) promoter region in HSC-3 cells. The values represented the means ± SD of at least three independent experiments. *p < 0.05 as compared with the vehicle group.

### Effects of *Selaginella* *tamariscina* on MAPK and Akt pathways

**Figure 6:**
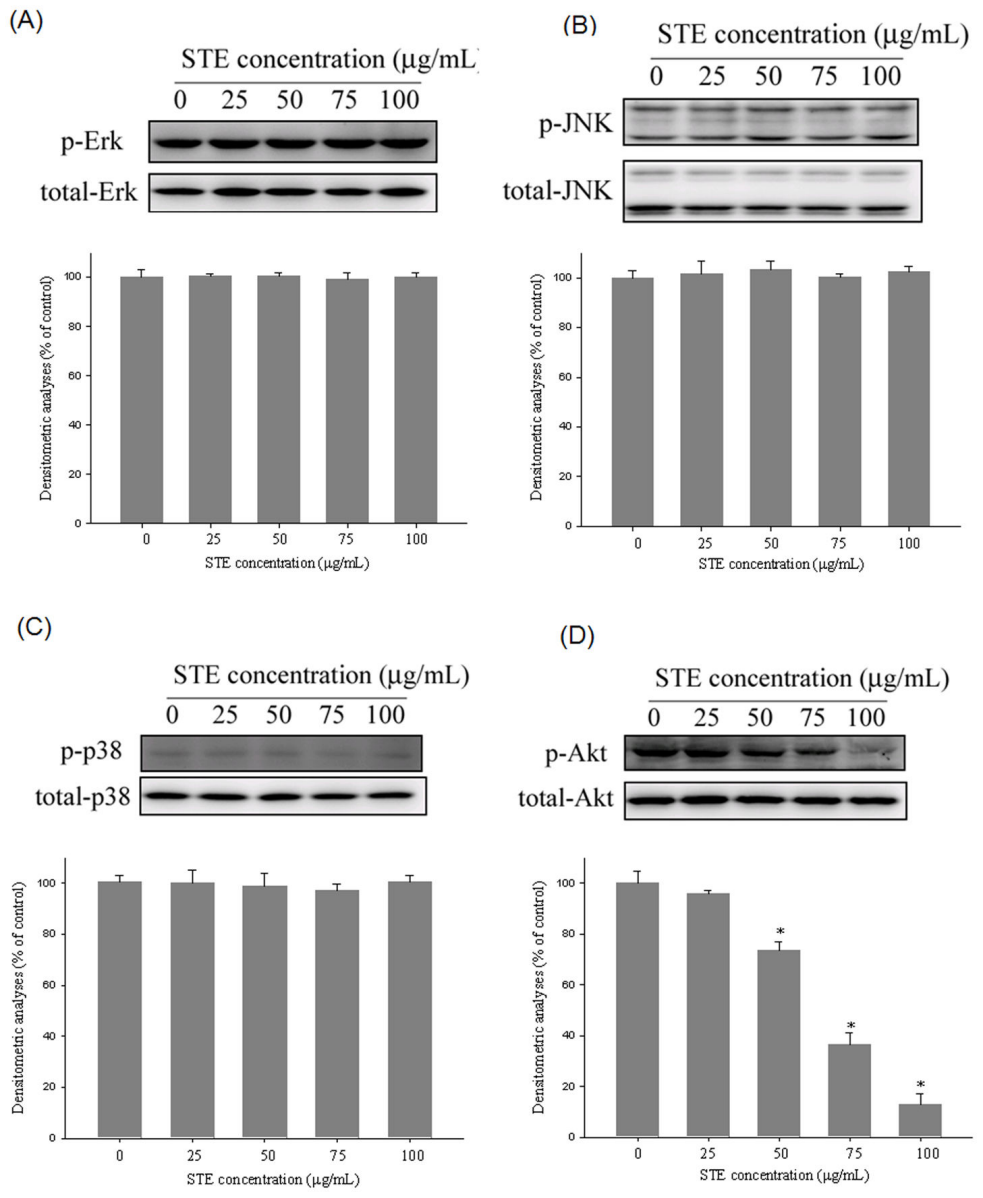
Effects of STE on the MAPKs pathway and Akt signalings.

To further investigate the underlying mechanisms of the upstream signaling pathways of MMP-2 and MMP-9, we used western blotting to evaluate the effects of *Selaginella* *tamariscina* on the MAPK and Akt pathways. Figure 6A–6C reveal that the MAPK pathway, which includes ERK, JNK, and p38 protein kinases, was not notably inhibited. However, *Selaginella* *tamariscina* reduced phosphorylation of Akt in a dose-dependent manner (Figure 6D). Thus, we suggest that the activation of the Akt signaling pathway is required for *Selaginella* *tamariscina* to suppress MMP-2 and MMP-9.

HSC-3 cells were cultured in various concentrations of STE (0, 25, 50, 75 and 100 µg/mL) for 24 hours, and then the cell lysates were subjected to SDS–PAGE followed by western blots with (A) anti-ERK1/2, (B) anti-JNK, (C) anti-p38 and (D) anti-Akt (total and phosphorylated) antibodies as described in Materials and Methods. Determined activities of these proteins were subsequently quantified by densitometric analyses with that of control being 100% as shown just below the gel data. The values represented the means ± SD of at least 3 independent experiments. *p< 0.05 as compared with the vehicle group.

## Discussion

Numerous medicinal plants have been studied for anticancer applications, such as *Dioscorea* *nipponica* Makino [23] and *Terminalia* Catappa [26]. Over the past decade, *Selaginella* *tamariscina* has become a traditional treatment for various diseases [14,15,18,19]. In this study, we suggest that *Selaginella* *tamariscina* exhibits beneficial effects on oral cancer cell treatment by (1) inhibiting HSC-3 oral cancer cell migration and invasion, (2) reducing MMP-2 and MMP-9 gene expression and enzyme activity, (3) inhibiting phosphorylation of AKT, (4) decreasing nuclear translocation of CREB and SP-1 to an MMP-2 promoter, and (5) decreasing nuclear translocation of AP-1 to an MMP-9 promoter. Numerous flavonoids are found in the crude extracts of *Selaginella* *tamariscina* that exhibit various pharmacological effects. Amentoflavone markedly arrested cell cycles and induced apoptosis of human breast and cervical cancer cells [21,27,28]. In addition, sumaflavone exerted anti-inflammatory effects by blocking iNOS expression through AP-1 inhibition [29]. Moreover, Mirzoeva et al showed that apigenin exhibits antiangiogenic potential in prostate carcinoma cells by inhibiting Smad2/3 and Src/FAK/Akt pathways [30]. The previous studies have suggested that flavonoids play a critical role in the anti-metastatic effects of *Selaginella* *tamariscina* , but the underlying mechanisms of this process require further explanation.

Metastasis, which causes approximately 90% of cancer deaths, is the process by which cancer cells spread from the original tumor site to distant organs [31]. The degradation of the ECM components and the basement membrane is a critical step in metastasis. There are multiple types of proteases that control ECM degradation and remodeling. MMP-2 and MMP-9 are the most extensively studied of the MMP family because of their high association with cancer migration and invasion [5]. Several previous studies have indicated that natural products inhibit cancer metastasis by inhibiting MMP-2 and MMP-9 expression [23,26]. Our results indicate that *Selaginella* *tamariscina* inhibited MMP-2 and MMP-9 enzyme activity, as well as protein expression. A decrease in migration and invasion abilities resulting from the suppression of MMP-2 and MMP-9 activity has been suggested. The results are similar to our previous study, in which the anti-metastatic effects of *Selaginella* *tamariscina* on lung cancer cells occurred through reduced gelatinase expression [19]. Numerous reports indicate that MMP gene expression was specifically regulated by mitogen-activated protein kinases (MAPKs), a family of serine/threonine kinases including ERKs, JNKs, and p38 [31–33]. However, our study results indicated that no observable effects on the MAPK signaling pathway resulted from the regulation of MMP production by *Selaginella* *tamariscina* . In addition, the involvement of the phosphoinositide-3 kinase (PI3K)/AKT signal transduction pathway in MMP gene expression and cell migration has been adequately studied. Wang et al revealed that isoliquiritigenin inhibited the expression and gelatinolytic activity of MMP-2 and MMP-9 by regulating the upstream AKT signaling pathways in breast cancer MDA-MB-231 cells [34]. Another study concluded that berberine, an isoquinoline alkaloid, inhibited breast cancer cell metastasis by modulating the AKT pathway [35]. Our data also suggested that the PI3K/AKT signaling pathway is involved as an upstream trigger of MMP-2 and MMP-9 regulation.

The expression of MMPs can be regulated at multiple levels, including transcription, post-transcription, translation, proenzyme-activation, and repression levels, by specific inhibitors [36]. It is suggested that *Selaginella* *tamariscina* regulated MMP-2 and MMP-9 at the transcriptional level because promoter activity and mRNA expression were inhibited. MMP promoters have several cis-elements that can be transactivated by several transcription factors, such as NF-κB, AP-1, CREB, and SP-1. Previous studies have indicated that the AKT/SP-1 pathway regulated MMP-2 promoter activity and affected the migration ability of cancer cells [24,37]. Satpathy et al showed that tissue transglutaminase 2 modulates CREB activation and MMP-2 transcription in ovarian cancer [38]. The upstream promoter sequence of the MMP-9 gene contains AP-1 and NF-κB sites. Epigallocatechin Gallate (EGCG) exerts its anti-invasive effect by suppressing AP-1 activation in human gastric cancer cells [39]. In addition, NF-κB regulates the expression of MMP-9 in various cancers [33,40,41]. Although MMP-9 mRNA expression was regulated by *Selaginella* *tamariscina* , we did not observe a notable effect on the NF-κB DNA-binding activities. Our study demonstrates that MMP-2 expression was regulated by CREB and SP-1 DNA-binding activities when affected by *Selaginella* *tamariscina* , and AP-1 site were necessary for the inhibition of MMP-9 expression.

The results of this study show that *Selaginella* *tamariscina* reduced oral cancer migration and invasion by inhibiting MMP-2 and MMP-9 gene expression, and enzyme activity. These anti-tumor effects on OSCC are associated with the suppression of AKT and the repression of DNA-binding activities on MMP-2 and MMP-9 promoters. OSCC invasion and metastasis are a major obstacle for cancer treatment. Therefore, the inhibition of metastasis by *Selaginella* *tamariscina* could provide vital preventive and therapeutic benefits for the treatment of oral cancer.
